# Effect of Hydrogen Sulfide on Sympathoinhibition in Obese Pithed Rats and Participation of K^+^ Channel

**DOI:** 10.1155/2024/5848352

**Published:** 2024-11-04

**Authors:** Carolina B. Gomez, Araceli Sánchez-López, Karla Carvajal, David Centurión

**Affiliations:** ^1^Departamento de Farmacobiología, Cinvestav-Coapa, Czda. de Los Tenorios 235, Col. Granjas-Coapa, Alc. Tlalpan, C.P. 14330, Ciudad de México, Mexico; ^2^Laboratorio de Nutrición Experimental, Instituto Nacional de Pediatría, Insurgentes Sur 3700 Letra C, Alc. Coyoacán, C.P. 04530, Ciudad de México, Mexico

**Keywords:** blood pressure, cardiovascular, high-fat diet, hydrogen sulfide, potassium channel

## Abstract

Elevated blood pressure is the leading metabolic risk factor in attributable deaths, and hydrogen sulfide (H_2_S) regulates vascular tone and blood pressure. Thus, this study aims to evaluate the mechanism by which NaHS (H_2_S donor) produces inhibition of the vasopressor sympathetic outflow in obese rats. For that purpose, animals were fed a high-fat diet (HFD) (60% calories from fat) for 12 weeks. They were anesthetized, pithed, and cannulated to evaluate the role of the potassium channel on NaHS-induced sympathoinhibition. Animals received selective electrical stimulation of the vasopressor sympathetic outflow, an intravenous (i.v.) administration of (1) tetraethylammonium (TEA, non-selective K^+^ channel blocker, 16.5 mg/kg), (2) 4-aminopyridine (4-AP, K_V_ channel blocker, 5 mg/kg), (3) barium chloride (BaCl_2_, K_IR_ channel blocker, 65 *μ*g/kg), (4) saline solution (vehicle of TEA, 4-AP, and BaCl_2_, 1 mL/kg), (5) glibenclamide (K_ATP_ channel blocker, 10 mg/kg), and (6) glibenclamide vehicle (DMSO + glucose 10% + NaOH, 1 mL/kg), and then a 310 *μ*g/kg·min NaHS i.v. continuous infusion. We observed that (1) NaHS produced inhibition of the vasopressor sympathetic outflow and (2) the sympathoinhibitory effect by NaHS was reversed by the K_IR_ channel blocker, BaCl_2_, in obese rats. The above data suggest that the potassium channel could be involved in the sympathoinhibition induced by NaHS.

## 1. Introduction

Obesity is a significant disease that affects individuals worldwide and is associated with excessive body fat accumulation, leading to weight gain and the development of chronic disorders, including arterial hypertension [1]. Obesity is associated with sympathoexcitation and hypertension [2], where the sympathetic nervous system is the primary contributor to the development and maintenance of hypertension [3]. It has been shown that the overflow of noradrenaline (NA) from peripheral vascular beds is higher, and the muscle sympathetic nerve activity is elevated in patients with hypertension compared to normotensive [3].

Previous findings have reported that during obesity, ATP-sensitive potassium channels (K_ATP_) controlling sympathetic nerve activity of brown adipose tissue are reduced [4]. In addition, a reduction in the expression of the K_ATP_ and the inward rectifier potassium channel (K_IR_) [5], calcium-activated potassium channel of intermediate conductance (IK_Ca_) [6], and voltage-gated potassium channel (K_v_) [7] has been associated with impaired vasodilatation in animal models of obesity.

Hydrogen sulfide (H_2_S) is a signaling molecule that contributes to the regulation of vascular tone [8] and blood pressure [9]. This effect is partially mediated by their effects on the vascular smooth muscle, endothelium, and sympathetic neurons [10]. Concerning this latter, it has been demonstrated that H_2_S modulates the activity of sympathetic neurons by activating K_ATP_ and K_IR_ [9]. A group evaluated the effect of H_2_S by recording the changes in the renal sympathetic nerve activity and found that exogenous H_2_S inhibits the sympathetic outflow in the carotid sinus. This was attributed to the opening of the K_ATP_ channel and the closing of the L-calcium channel [11]. Also, Dominguez [12] revealed that H_2_S reduced the K^+^ currents in trigeminal neurons involving K_v_7. In addition, we have previously reported that H_2_S inhibited the vasopressor sympathetic outflow when preganglionic stimulation is performed [13] by activation of several potassium channels, including K_ATP_, K_IR_, K_V_, and BK_Ca_ (large-conductance calcium- and voltage-activated K^+^) channel [14].

In a previous study performed in rats with obesity induced by a high-fat diet (HFD), it was demonstrated that chronic treatment with sodium hydrosulfide (NaHS) was effective in ameliorating the cardiovascular changes induced by obesity; this means reducing the vasopressor sympathetic outflow [15]. Thus, NaHS effectively reverses the cardiovascular changes induced by metabolic diseases. Further, hydrogen sulfide production is reduced in overweight subjects compared to lean volunteers [16] and reduced CSE (cystathionine g-lyase) or CBS (cystathionine-b-synthase) enzyme activity in obese rats [17]; meanwhile, supplementation with hydrogen sulfide donors such as garlic [18], DATS [19], or NaHS [15] reduced body weight gain in obese rats.

The sympathetic nervous system regulates blood pressure by influencing vasculature, kidneys, and the heart. One of the reasons for obesity-induced hypertension is sympathoexcitation [20]. Thus, it can be suggested that H_2_S may play an essential role in regulating the excitability of sympathetic fibers. This can be done by modulating potassium channel activity, although the potential mechanisms to produce this effect in obesity are unknown. Thus, this study aimed to explore in HFD-induced obese rats (1) the potential sympathoinhibitory effect of NaHS and (2) the role of several potassium channels in this effect.

## 2. Methodology

### 2.1. Animals

138 male Wistar rats weighting 200–250 g provided by the institutional laboratory animal facility were housed in acrylic cages, maintained under standardized conditions (22 ± 2°C, 50% humidity, and a 12/12 h light-dark cycle), and provided with food (control or HFD) and water *ad libitum*.

### 2.2. Diet

All the animals were divided into two groups: (1) fed with a control diet LabDiet 5008 and (2) fed with an HFD (63% of calories provided by fat; made with LabDiet 5008 powder mixed with hazelnut cream and lard) for 12 weeks. This time is enough to induce obesity alterations such as hypertension and an increase in vasopressor responses induced by sympathetic stimulation, as previously reported by Gomez et al. [15].

### 2.3. Determination of Arterial Blood Pressure and Heart Rate (HR) in Conscious Animals

We measured arterial blood pressure and HR by a tail-cuff method using a LE 5001 automatic blood pressure recorder (Letical, PanLab, Barcelona, Spain). For this purpose, the animals were habituated for five days before the measurements. Thus, all animals were immobilized for a short period in acrylic cages, and their tails were left exposed to a heating lamp for 15 min. Afterward, the cuff was placed in the tail and inflated to stop blood flow. Then, the tension released allowed us to determine systolic blood pressure (SBP) through the sensor and calculate HR, diastolic blood pressure (DBP), and mean blood pressure (MBP). The measurement was done at 12 weeks, representing the end of the HFD period.

### 2.4. Evaluation of Cardiovascular Function

At the end of the HFD period, animals were anesthetized with isoflurane (3%), and the trachea was cannulated to provide them with artificial ventilation. The animals were artificially ventilated with room air using a positive pressure pump (7025 rodent ventilator, Ugo Basile, Comerio, VA, Italy) at 80 strokes/min and a 20 mL/kg stroke volume, as previously described [21]. Animals were pithed by inserting a stainless-steel rod through the orbit and *foramen magnum* into the *vertebral foramen*, intending to destroy the central nervous system [22], and we performed a bilateral vagotomy. This procedure was performed to avoid the influence of the central nervous system over the hemodynamic variables and responses induced by the analyzed compounds. This can be confirmed by observing the DBP in 40–50 mm·Hg compared to an anesthetized rat [23].

After a bilateral vagotomy, catheters were placed in (1) the left carotid artery to record arterial blood pressure and HR with a pressure transducer (RX104A, Biopac Systems Inc., Goleta, CA, USA), (2) the right femoral vein for drug i.v. administration (potassium channel blockers), and (3) the left femoral vein for drug infusion (NaHS). Arterial blood pressure and HR were recorded simultaneously using a data acquisition unit (MP150A-CE, Biopac Systems Inc., Goleta, CA.). DBP was determined, as is the pressure when the left ventricle is relaxed and thus could indirectly represent the systemic vascular resistance that regulates arterial blood pressure and blood flow within organs. Pithed rats were maintained at a body temperature of 37°C by a lamp and monitored with a rectal thermometer.

### 2.5. Experimental Design in Pithed Rats

**Figure 1:**
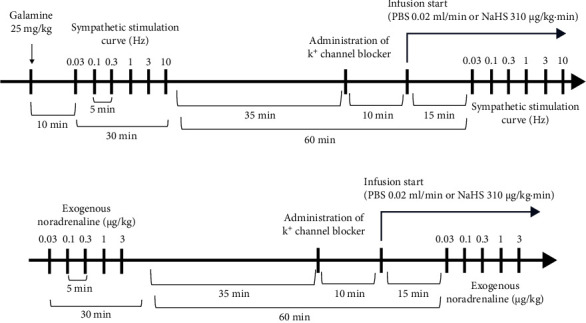
Timeline of sympathetic stimulation, stimulation with exogenous noradrenaline, and drug administration.

All rats followed the following protocol after the procedure mentioned above. First, we determined the vasopressor responses induced by selective preganglionic (T_7_–T_9_) stimulation of the vasopressor sympathetic outflow. For this objective, a stainless-steel rod was replaced by an enameled bipolar electrode except for a 1 cm length 9 cm from the tip. The uncovered segment of the bipolar electrode was placed at the T_7_–T_9_ region of the spinal cord, allowing the selective stimulation of the sympathetic nerves that supply the systemic vasculature [24], ensuring that arterial pressure but not HR changes at stimulation. To avoid electrically induced muscle twitching, animals received an i.v. bolus of gallamine (a non-depolarizing muscle relaxant, 25 mg/kg) before the electrical stimulation. The preganglionic vasopressor sympathetic outflow was stimulated with an S88X square pulse stimulator (Grass Technologies, Warwick, RI, USA) by applying 10 s trains of monophasic, rectangular pulses (2 ms, 60 V) at increasing frequencies (0.03, 0.1, 0.3, 1, 3, and 10 Hz). Once the stimuli–response curve was completed, the animals were separated into three main groups to evaluate (1) the sympathoinhibitory effect of NaHS, (2) the per se effect of K^+^ channel blockers, and (3) the effect of K^+^ channel blockers on the sympathoinhibition produced by NaHS (Figure 1).

### 2.6. Evaluation of the Sympathoinhibitory Effect of NaHS

**Figure 2:**
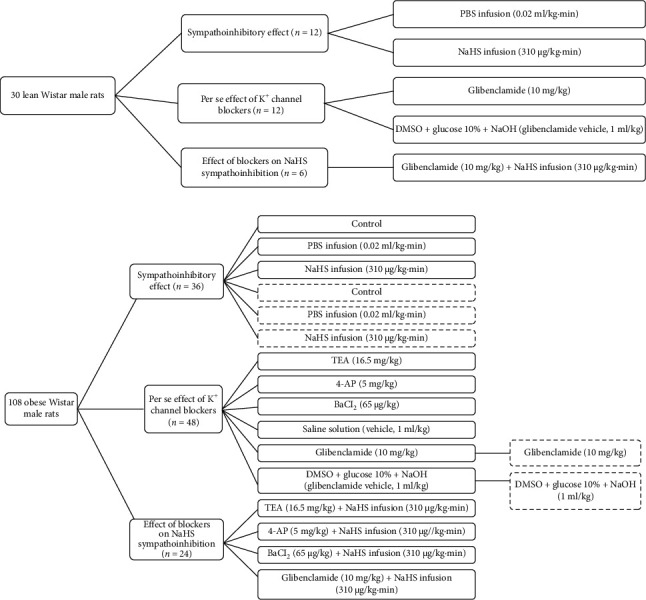
Experimental design. Solid line: electrical stimulation. Dotted line: exogenous noradrenaline.

This group included 48 animals and was divided into two subgroups. The first subgroup (*n* = 12) were lean rats, and the second (*n* = 36) were obese rats. Then, these were divided into different sets (*n* = 6 each) where animals received an i.v. infusion of (1) nothing (control group) where animals received a second stimuli–response curve as mentioned above, (2) PBS (0.02 mL/kg·min), or (3) NaHS (310 *μ*g/kg·min) before the second stimuli–response curve was performed (Figure 2). In the second subgroup (*n* = 6 each), the effect of i.v. bolus injections of NA (0.03–3 mg/kg) was determined before (control) and during i.v. infusion of (1) nothing (control group), (2) PBS (0.02 mL/kg·min), and (3) NaHS (310 *μ*g/kg·min) (Figure 2(b)). We choose 310 *μ*g/kg·min NaHS because it has an inhibitory effect without significantly modifying baseline values of DBP [13].

### 2.7. Evaluation of per se Effect of K^+^ Channel Blockers

In this case, obese animals (*n* = 48) were divided into two main groups: (1) electrically stimulated and (2) stimulated with exogenous NA. The first group (*n* = 36) was divided into six subgroups (*n* = 6 each) to evaluate the per se effect of several K^+^ channel blockers and their vehicles by i.v. administration: (1) TEA (non-selective K^+^ channel blocker, 16.5 mg/kg), (2) 4-AP (K_V_ channel blocker, 5 mg/kg), (3) barium chloride (BaCl_2_, K_IR_ channel blocker, 65 *μ*g/kg), (4) saline solution (vehicle of TEA, 4-AP and BaCl_2_, 1 mL/kg), (5) glibenclamide (K_ATP_ channel blocker, 10 mg/kg), and (6) glibenclamide vehicle (DMSO + glucose 10% + NaOH, 1 mL/kg). Twenty-five minutes after the i.v. administration of these blockers, the second stimuli–response curve was performed (Figure 1). The second group (*n* = 12) was divided into two subgroups (*n* = 6 each): (1) glibenclamide (10 mg/kg) and (2) glibenclamide vehicle (1 mL/kg) (Figure 2(b)). Additionally, we included two groups (*n* = 6 each) of lean rats: (1) glibenclamide (10 mg/kg) and (2) glibenclamide vehicle (1 mL/kg).

### 2.8. Evaluation of the Effect of K^+^ Channel Blockers on the Sympathoinhibition Produced by NaHS

For this objective, obese animals (*n* = 24) were divided into four subgroups (*n* = 6) that received an i.v. administration of (1) TEA (16.5 mg/kg), (2) 4-AP (5 mg/kg), (3) BaCl_2_ (65 *μ*g/kg), and (4) glibenclamide (10 mg/kg) (Figure 2(b)), and 10 min after, the NaHS infusion was initiated. Fifteen minutes after the initiation of the infusion, the second stimuli–response curve was performed (Figure 1(a)). At last, a group (*n* = 6) of lean rats received an i.v. administration of glibenclamide (10 mg/kg) with the NaHS continuous infusion (Figure 2(a)).

### 2.9. Drugs

In addition to the anesthetic isoflurane (Floriso, Vet Ones, Boise, ID, USA), the compounds used in this study were gallamine triethiodide (PubChem CID: 6172), sodium hydrosulfide monohydrate (PubChem CID: 28015), TEA chloride (PubChem CID: 5946), 4-AP (PubChem CID: 1727), barium chloride (PubChem CID: 25204), glibenclamide (PubChem CID: 3488), and NA (PubChem CID: 53399078).

### 2.10. Statistical Analysis

All results in the figures are presented as mean ± S.E.M. The maximum changes in DBP due to electrical stimulation were determined and measured before and after administering vehicles, K^+^ channel blockers and i.v. NaHS, or vehicle infusions. A Tukey's test evaluated the difference between the changes in DBP within the groups of animals once the two-way repeated measure analysis of variance reached statistical significance. Statistical significance was accepted at *p* < 0.05.

## 3. Results

As already reported by several studies, HFD causes hepatic steatosis [25], obesity-related dysbiosis [26] as well as cardiotoxicity [27], and an increase in hemodynamic (blood pressure and HR), zoometric (weight), and metabolic variables (glucose, insulin, leptin, and ghrelin) [15]. In this study, at the end of the 12 weeks of diet, lean animals weighed an average of 479 ± 9 g and obese rats 561.50⁣ ± ⁣46.18^∗^ g, which is significantly different (*p* < 0.05). Related to the hemodynamic variables, we found the following measures in lean rats: 354 ± 20 bpm for HR, 114 ± 5 mm Hg SBP, 86 ± 6 mm Hg DBP, and 96 ± 5 mm Hg MBP and in obese rats: 393 ± 18 bpm for HR, 163⁣ ± 5^∗^ mm·Hg SBP, 128⁣ ± 13^∗^ mm·Hg DBP, and 140⁣ ± 5^∗^ mm·Hg MBP (∗, *p* <  0.05 compared to lean rats).

### 3.1. Sympathoinhibitory Effect of NaHS

**Figure 3:**
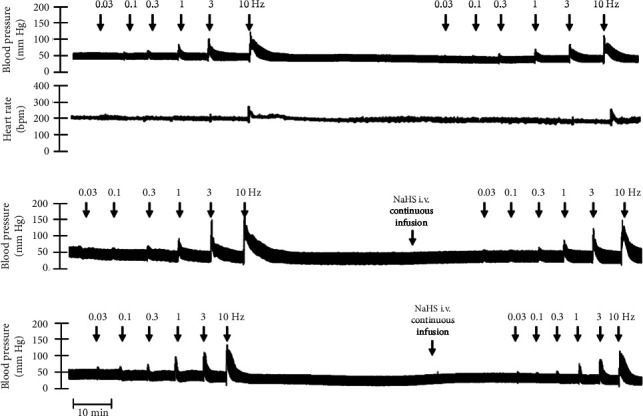
Original recordings of NaHS-induced sympathoinhibition in lean and obese rats. Original recordings show the vasopressor responses induced by selective electrical stimulation of the vasopressor sympathetic outflow at the frequencies of 0.03, 0.1, 0.3, 1.0, 3.0, and 10 Hz in the absence (a) or the presence (b, c) of i.v. infusion of 310 *μ*g/kg·min NaHS in lean (b) and obese (c) rats. Also, no significant changes in heart rate were observed during the electrical stimulation.

**Figure 4:**
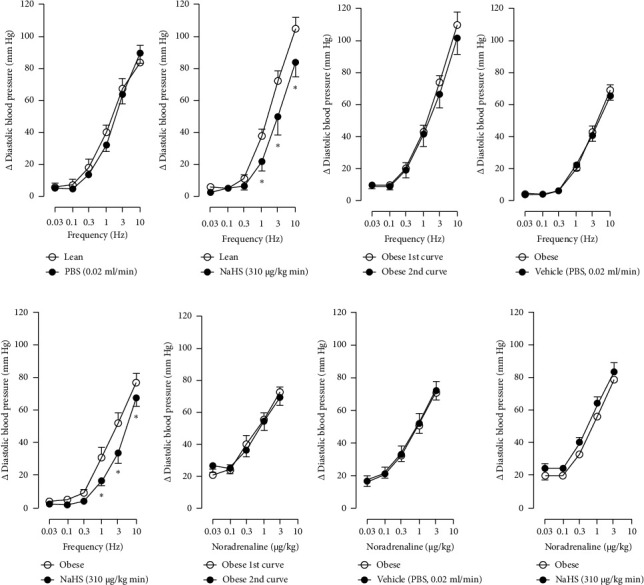
Effect of continuous i.v. infusion of PBS and NaHS on the vasopressor responses induced by sympathetic stimulation in lean (a, b) and obese (c–e) rats or exogenous noradrenaline in obese rats (f–h). Each point represents the mean ± S.E.M of 6 animals. ⁣^∗^, *p*  <  0.05 vs. “lean” or “obese,” respectively.

The first step in our investigation was to establish that the infusion of NaHS would inhibit the sympathetic system in lean and obese rats. In Figure 3, we show an original trace of the vasopressor responses induced by selective electrical stimulation of the sympathetic vasopressor outflow in lean and obese animals. It can be observed in Figure 3(a) that electrical stimulation of the sympathetic outflow produced increases in blood pressure with negligible changes in HR. Thus, as seen in Figure 4, we first evaluated the reproducibility of the vasopressor responses induced by electric stimulation with infusion of PBS (Figure 4(a)) and NaHS (Figure 4(b)) in lean rats, finding an inhibition in the vasopressor responses in the curve with NaHS infusion. In Figure 4(c), we evaluated the reproducibility of the vasopressor responses over time to prove that time alone did not affect the vascular responses, with an hour difference between each curve. Next, the responses under the infusion of vehicle (PBS, Figure 4(d)) or NaHS (Figure 4(e)) were evaluated this time in obese rats. In the latest, the infusion with NaHS, but not PBS, significantly diminished the vasopressor responses obtained compared with the control curve. This result can lead us to conclude that NaHS produces a sympathoinhibitory effect in both lean and obese rats. In addition, we calculated the area under the curve (AUC) of the vasopressor responses induced by sympathetic stimulation in lean and obese rats. Interestingly, the AUC in obese rats (504.83⁣ ± ⁣32^∗^) was significantly lower than the AUC in lean rats (699 ± 43, ⁣^∗^, *p*=0.002). These results suggest that a decrease in the vasopressor response was established in obese rats. We also calculated the AUC for Figure 4(b) (lean + NaHS) vs. Figure 4(e) (obese + NaHS), obtaining the following values, 549 ± 86 and 411.13 ± 45, respectively. These values were not significantly different, indicating that the sympathoinhibitory effect in lean rats was similar to that observed in obese rats. Nevertheless, to ascertain this result, a NA curve was performed in obese rats as NA is an *α*-adrenergic agonist; the following experiment would eliminate the possibility of a direct effect of NaHS on the blood vessels. As seen in Figure 4(f), we evaluated the reproducibility of the vasopressor responses induced by exogenous NA in the same animal, with an hour difference between each curve, proving that time did not affect the responses. In Figures 4(g) and 4(h), we can observe that i.v. infusion of NaHS did not significantly modify the vasopressor responses induced by exogenous NA, implying that NaHS acts on the sympathetic tone.

### 3.2. Per se Effect of K^+^ Channel Blockers on the Vasopressor Responses to Sympathetic Stimulation

**Figure 5:**
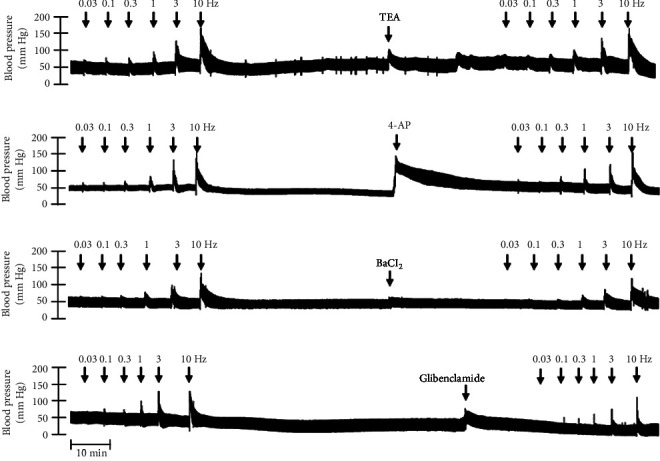
Original recordings of K^+^ channel blockers in obese rats. Original recordings show the vasopressor responses induced by selective electrical stimulation of the vasopressor sympathetic outflow at the frequencies of 0.03, 0.1, 0.3, 1.0, 3.0, and 10 Hz in the presence of i.v. administration of (a) TEA (16.5 mg/kg), (b) 4-AP (5 mg/kg), (c) BaCl_2_ (65 *μ*g/kg), and (d) glibenclamide (10 mg/kg).

**Figure 6:**
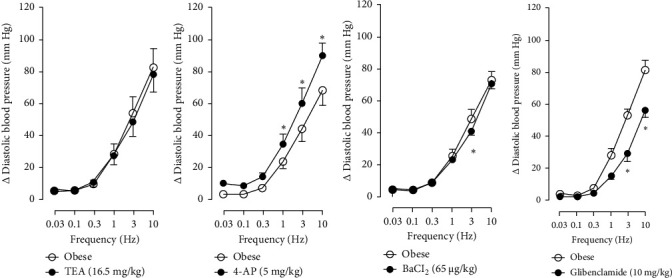
Effect of (a) TEA (16.5 mg/kg), (b) 4-AP (5 mg/kg), (c) BaCl_2_ (65 *μ*g/kg), and (d) glibenclamide (10 mg/kg) over the vasopressor responses induced by electrical stimulation of the sympathetic tone in obese rats. Each point represents the mean ± S.E.M of 6 animals. ⁣^∗^, *p*  <  0.05 vs. obese.

In Figures 5 and 6, we can observe the per se effect of the K^+^ channel blocker in the original recording and curve graphics, respectively, in obese rats. Firstly, the impact of TEA was evaluated. In Figure 6(a), the effect per se of the blocker shows that the frequency-response curve did not significantly change compared to the control curve. Secondly, 4-AP, a blocker for the K_V_ channel (Figure 6(b)), significantly increased the vasopressor responses induced by electrical stimulation, particularly at the frequencies of 1, 3, and 10 Hz. Thirdly, Figure 6(c) shows the effect of BaCl_2_ (K_IR_ channel blocker). As observed, BaCl_2_ failed to modify the vasopressor responses induced by sympathetic stimulation, except at 3 Hz, as we observed a significative reduction. Lastly, the effect of glibenclamide on the vasopressor responses induced by stimulation of the vasopressor sympathetic tone is shown in Figure 6(d). In this respect, glibenclamide per se induced a significant decrease in the vasopressor responses, especially at the frequencies of 3 and 10 Hz.

### 3.3. Effect of Several K^+^ Channel Blockers on the Sympathoinhibitory Effect of NaHS

**Figure 7:**
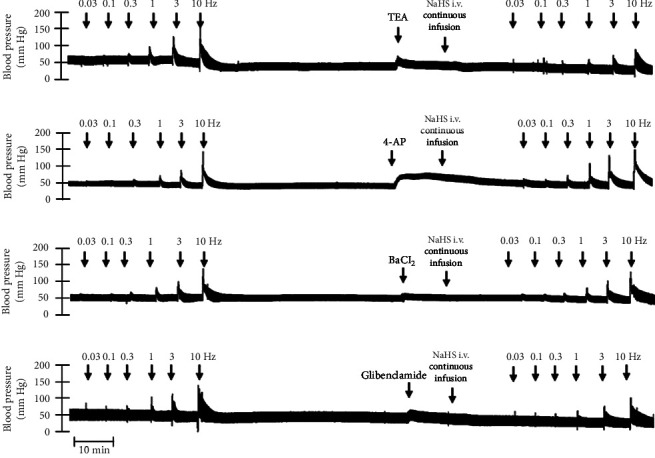
Original recordings of the effect of K^+^ channel blockers and NaHS i.v. continuous infusion in obese rats. Original recordings show the vasopressor responses induced by selective electrical stimulation of the vasopressor sympathetic outflow at the frequencies of 0.03, 0.1, 0.3, 1.0, 3.0, and 10 Hz in the presence of i.v. administration of (a) TEA (16.5 mg/kg), (b) 4-AP (5 mg/kg), (c) BaCl_2_ (65 *μ*g/kg), and (d) glibenclamide (10 mg/kg) and the continuous i.v. infusion of NaHS (310 *μ*g/kg·min).

**Figure 8:**
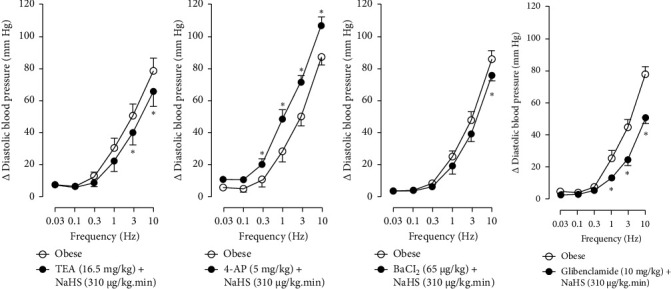
Effect of (a) TEA (16.5 mg/kg), (b) 4-AP (5 mg/kg), (c) BaCl_2_ (65 *μ*g/kg), and (d) glibenclamide (10 mg/kg) over the sympathoinhibition induced by NaHS infusion (310 *μ*g/kg·min). Each point represents the mean ± S.E.M of 6 animals. ⁣^∗^, *p*  <  0.05 vs. obese.

**Figure 9:**
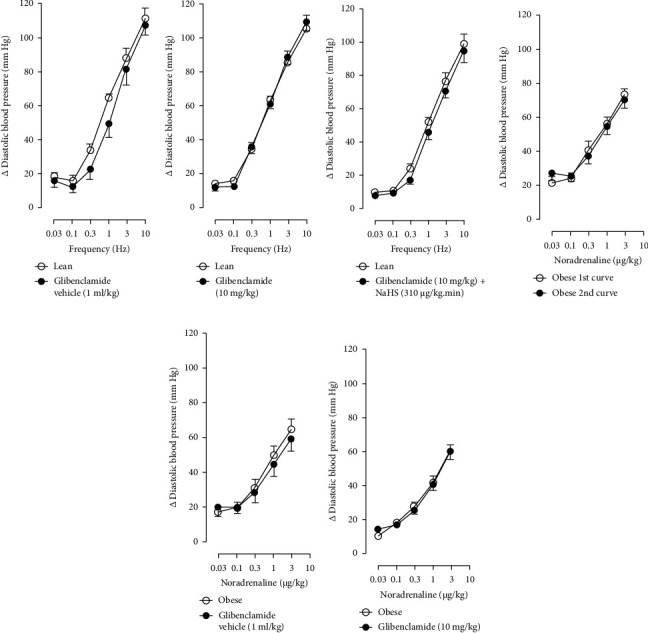
Effect of (a) glibenclamide vehicle (control), (b) glibenclamide (10 mg/kg), and (c) glibenclamide (10 mg/kg) + NaHS infusion (310 *μ*g/kg·min) on the vasopressor responses induced by electrical stimulation in lean rats. Effect of (d) glibenclamide vehicle (control), (e) glibenclamide (10 mg/kg), and (f) glibenclamide (10 mg/kg) + NaHS infusion (310 *μ*g/kg·min) on the vasopressor responses induced by exogenous noradrenaline in obese rats. Each point represents the mean ± S.E.M of 6 animals.

Figures 7 and 8 show the effect of several K^+^ channel blockers on the sympathoinhibitory effect of NaHS. Thus, TEA partially blocks the inhibition caused by NaHS (Figure 8(a)), namely, at 1 Hz (compared with Figure 6(a)). This can be interpreted as that any K^+^ channel is one of the various mechanisms so that we evaluate specific K^+^ channel blockers to elucidate which ones could be involved in the sympathoinhibitory effect of NaHS. In the case of 4-AP, the enhancement induced by this drug per se (Figure 6(b)) was maintained during the infusion of NaHS (in Figure 8(b)) at the frequencies 0.3–10 Hz. BaCl_2_ blocks the inhibition produced by NaHS at 1 and 3 Hz (Figure 8(c)). With this result, the sympathoinhibitory effect of NaHS is partially mediated through the K_IR_ channel. Lastly, as shown in Figure 8(d), the sympathoinhibition to NaHS at 1, 3, and 10 Hz frequencies remained unaffected in the presence of glibenclamide. As glibenclamide produced a per se inhibition of the vasopressor responses to sympathetic stimulation (Figure 6(d)), we decided to evaluate the effect of glibenclamide on the vasopressor responses to exogenous NA in obese rats and to electrical stimulation in lean rats. Thus, Figures 9(a)–9(c) demonstrate that glibenclamide or the respective vehicle did not modify the vasopressor responses induced by electrical stimulation in lean rats, and or the vasopressor responses induced by exogenous NA administration, see Figures 9(d)–9(f), in the vasopressor responses induced by exogenous NA administration. These results suggest that glibenclamide could inhibit the vasopressor responses induced by sympathetic stimulation through a prejunctional mechanism.

## 4. Discussion

Since the first report of H_2_S as a neuromodulator [28], multiple beneficial effects of H_2_S have been reported in the body, including metabolic effects such as (1) an increase of GLUT4 protein levels and translocation to increase glucose uptake and utilization in adipose tissue, (2) a decrease of FoxO1 in the liver to decrease gluconeogenesis, and (3) a decrease of reactive oxygen species in muscle [29]. We have previously reported that HFD increased the hemodynamic variables such as blood pressure and HR, while the four weeks of treatment with NaHS reversed hypertension [15]. Subsequently, Medina-Terol et al. [14] concluded that H_2_S mediates sympathoinhibition by activating several K^+^ channels in lean rats. Despite the above findings, little is known about the mechanism of the sympathoinhibition caused by NaHS in obese rats.

In a previous study, our research group demonstrated that NaHS can inhibit sympathetic transmission in systemic vasculature in lean animals [13]. The pithed rat model allows us to evaluate the sympathetic tone indirectly, as we can measure the neurotransmitter release in the sympathetic fibers. When electrical stimulation occurs at the level of sympathetic fibers that stimulate blood vessels, NA is released from the sympathetic terminal, which binds to the a-adrenergic receptors of the blood vessels and produces contraction [30]. This helps us explain the reason why NaHS can produce a diminishment of vasoconstriction produced by electrical stimulation; when we use NA to induce vasoconstriction, no effect of NaHS was observed (Figure 4). This result led us to conclude that NaHS produced a sympathoinhibitory effect. Although we do not have a clear-cut explanation in the differences of the vasopressor responses induced by stimulation of the sympathetic outflow between lean and obese rats, a reduction in the responses may be due to a decrease in NA release, downregulation of *α*-adrenoceptors, and/or a decrease in the signaling pathways activated by *α*-adrenoceptors.

When comparing with the results obtained by Medina-Terol et al. [14] in lean animals, we can summarize that (1) NaHS induces sympathoinhibition at the frequencies of 1, 3, and 10 Hz (Figure 2(c)) in obese and lean rats; (2) per se effect of 4-AP increased vascular responses to electrical stimulation (Figure 6(b)) in obese and lean rats, and (3) BaCl_2_ blocked the sympathoinhibition induced by NaHS at the frequencies of 1 and 3 Hz, in lean and obese animals (Figure 8(c)). On the other hand, and what is worth highlighting is the difference in the effect to glibenclamide on the sympathoinhibition to NaHS. In lean animals, glibenclamide did not produce a per se effect. When administered with NaHS infusion, it could block the sympathoinhibition induced by NaHS ([14] and Figure 9(c)). In contrast, in obese rats, we found that (1) glibenclamide per se decreased the vasopressor responses induced by electrical stimulation at the frequency of 3 and 10 Hz (Figure 6(d)) and (2) glibenclamide did not block the sympathoinhibition induced by NaHS at any level (Figure 8(d)). Thus, in obese rats, it is possible that HFD induces a functional modification in K_ATP_ channel that deserves further investigation.

At this point, the doses of the K^+^ channel blockers were carefully selected based on previous investigations. For example, 16.5 mg/kg TEA significantly reduced the responses to NaHS [14, 31]. Glibenclamide is a selective blocker of the K_ATP_ channel; it has been observed that the 10 mg/kg dose can block the vasodepressor responses induced by diazoxide, a K_ATP_ activator [32]. Berg and Koteng [33] demonstrated that 5 mg/kg 4-AP can block the bradykinin-induced vasodepressor responses. Finally, 65 *μ*g/kg BaCl_2_ is a dose that can block renal vasodilation in anesthetized rats [34].

As TEA is a non-selective K^+^ channel blocker, the results obtained allowed us to inquire more about which K^+^ channel is involved in the sympathoinhibitory effect of NaHS. K^+^ channels regulate neurotransmitter release, allowing K^+^ to cross the membrane in sympathetic nerve fibers. Four K^+^ channel families have been reported, including K_v_, K_IR_, K_Ca_, and K_2P_ (two-pore domain potassium) [35]. When evaluating the per se effect of the K^+^ channel blockers, we found that the administration of 4-AP, a K_v_ channel blocker, produced an increase in the vasopressor responses induced by sympathetic stimulation (Figure 4(b)). This is probable due to the blockade of the repolarizing currents by channels localized in the sympathetic terminal. Indeed, this blockade is translated into a longer duration of the action potential; therefore, a more significant entry of calcium in the sympathetic terminal and an increase in released neurotransmitters to the neuroeffector junction could be induced by TEA. There is evidence that high NaHS concentrations did reduce currents through the K_v_7 channel, and this mechanism must be expected to depolarize the neurons slightly and increase action potential firing [36]. Also, Dominguez-Rodriguez et al. [12] revealed that H_2_S reduced the K^+^ currents in trigeminal neurons involving K_v_7. As per the result found in Figure 8(b), it could be possible that NaHS produced a persulfidation of K_v_ channels, a post-translational modification of proteins, and may change the function of the channel to depolarization, as suggested by Velleco et al. [37].

By looking at the effect of K^+^ channel blockers on sympathoinhibition, we found that BaCl_2_ reduced the sympathoinhibition produced by NaHS (Figure 6(c)). In this respect, it has been suggested that H_2_S can interact with the 43-cysteine residue of the K_IR_6.1 subunit of K_ATP_ channel [38]; persulfidation of these residues can generate a conformational change in the channel structure that allows its opening [39]. An output of K^+^ is generated that induces the hyperpolarization of the membrane of the sympathetic fibers, causing the closure of the Ca^2+^ channel, and, in this way, the release of NA vesicles is decreased. In this sense, the decrease in NA release is reflected as a decrease in vasopressor responses induced by electrical stimulation of the sympathetic outflow.

In the case of the K_ATP_ channel, we obtained an exciting result in obese rats. As seen in blood vessels, the opening of K_ATP_ channels induces vasorelaxation and, blocking these channels, vasoconstriction [40]. Further, in guinea pig and human isolated right atrium, K_ATP_ activation reduces neurotransmitter release [41]. Salvi et al. [42] reported that glibenclamide reverses the inhibition of evoked NA release induced by an H_2_S donor, concluding that H_2_S donors can inhibit the sympathetic neurotransmission in isolated bovine iris-ciliary bodies mediated by the action on the K_ATP_ channel. Also, it was found that a preinjection of glibenclamide abolished the sympathoinhibitory effects of NaHS; this suggests that exogenous H_2_S in the rostral ventrolateral medulla inhibits sympathetic vasomotor tone by the opening of K_ATP_ channel [43]. This evidence contradicts our findings as the administration of glibenclamide per se, and in the presence of NaHS, produced a diminishment of the vasopressor responses induced by sympathetic stimulation (Figure 6(d)), which may suggest a sympathoinhibition in obese rats. For this reason, we performed another experiment in aged-matched lean animals (Figure 9) where the vehicle (Figure 9(a)), glibenclamide (Figure 9(b)), and glibenclamide plus the NaHS i.v. continuous infusion (Figure 9(c)) did not cause any effect and both curves are the same. Then, the effect of glibenclamide was determined before and after the administration of exogenous NA to verify that the observed effect did not occur in the blood vessels and the K^+^ channel located in them. In this way, NA stimulates *α*-adrenoceptors in the vascular smooth muscle, and glibenclamide did not modify the vasopressor responses into NA (Figures 9(c)–9(f)). Therefore, under our experimental conditions: (1) glibenclamide per se can inhibit the sympathetic neurons by a prejunctional mechanism not identified and (2) hydrogen sulfide probably stimulates a prejunctional mechanism non-related to the K_ATP_ channel. In this respect, (1) glibenclamide can interact with several other molecular targets including cystic fibrosis transmembrane conductance regulator Cl^−^ channels (CFTR-Cl) [44], and peptide transporters 1 and 2 (PEPT1 and PEPT2) [45] and (2) reduction in KATP-mediated dilation, KATP currents, and KATP expresion in vascular smooth muscle cells were observed by Fan [46] in an experimental model of diet-induced obesity, ATP, impliying a reduction in the function of the K_ATP_ channel in obesity. This leads to the conclusion that an HFD could increase the expression or function of K_ATP_ channels in sympathetic fibers. As in a previous study carried out in lean rats, the administration of glibenclamide plus the NaHS infusion did not cause any effect on the vasopressor responses [14]. We hypothesize that K_ATP_ channels in the HFD animals are dysfunctional, which deserves further exploration. Consistent with the above, it has been demonstrated that obesity caused an increase in blood pressure, downregulation of K_ATP_ expression, and a diminishment of K_ATP_ channel–mediated relaxation responses in blood vessels as free fatty acids could inhibit K_ATP_ channel and the NO produced by endothelium was also inhibited by HFD [5]. In another study where rats were given a high-fat and high-fructose diet for 28 weeks, a reduction in the mRNA expression encoding different potassium channels like SK_Ca_, IK_Ca_, and K_IR_2.1 was detected, which was associated with an increase in SBP and dilatation in small arteries [6]. Another study reported a rise of K_v_ channel activity in hypertensive vascular smooth muscle [47], as a reduction of vascular K_v_ channel function has been confirmed in arteries and arterioles of animals from different models as diet-induced hypercholesterolemia, metabolic syndrome, and obesity [48–50]. We also know that glucose metabolism increases the ATP/ADP ratio, leading to the closure of the K_ATP_ channel and cell depolarization. More recently, Fisher [51] investigated if the K_ATP_ channel activity or expression was altered in a mouse model of HFD-induced obesity. They found that the expression of two K_ATP_ channel subunits was decreased in the central and peripheral nervous system in mice in this obesity model. This led to a need for more studies about how our habits led to structural and molecular changes like ion channels in the nervous system [51].

So, the results in this study give us an idea of the following experiments to perform in obese rats and the alterations in the K^+^ channel in fibers that occur during obesity. The neuronal K_ATP_ channel contains a K_IR_ 6.2 subunit. K_ATP_ channels respond to adenine nucleotide fluctuations at intracellular levels and are inhibited by ATP. The ability to sense ATP/ADP levels ensures that the changes produced in cellular metabolism can be translated to changes in the membrane K^+^ permeability, which translates to changes in membrane potential and excitability [35]. As known, ATP production is elevated in insulin-sensitive cells under obese conditions [52]. This could also be a mechanism by which we are finding this channel activity. Admittedly, we cannot categorically exclude the participation of K_ATP_ channels as glibenclamide inhibited, per se, the vasopressor responses to electrical stimulation.

Altogether, we demonstrate that K^+^ channels are involved in the mechanism by which NaHS induces sympathoinhibition in obese rats, although the mechanisms involved differ from those in lean rats. Nevertheless, more research should be carried out in this regard and if there is any modification on the K^+^ channel localized at prejunctional fibers induced by HFD.

## 5. Conclusion

In conclusion, HFD-induced obesity modifies the sympathoinhibition to H_2_S, as K_IR_ channels are involved in this effect. These findings may be clinically relevant since (1) during obesity, plasma levels of hydrogen sulfide are reduced [16] and (2) administration of hydrogen sulfide donors could reduce sympathoexcitation observed in obesity. Thus, this encourages us to perform more investigations on exploring the participation of K_ATP_ channels by using other pharmacological tools, molecular, electrophysiological, imaging techniques, or mass spectrometry to detect protein persulfidation.

## Data Availability

The raw data used to support the findings of this study are available from the corresponding author upon request.
